# Acute Urinary Retention During Pregnancy—A Nationwide Population-Based Cohort Study in Taiwan

**DOI:** 10.1097/MD.0000000000003265

**Published:** 2016-04-01

**Authors:** Jeng-Sheng Chen, Solomon Chih-Cheng Chen, Chin-Li Lu, Hsin-Yi Yang, Panchalli Wang, Li-Chung Huang, Fu-Shun Liu

**Affiliations:** From the Department of Urology, Sinying Hospital, Ministry of Health and Welfare, Sinying (J-SC); Department of Medical Research (C-LL, H-YY, SC-CC), Ditmanson Medical Foundation Chia-Yi Christian Hospital, Chia-Yi; Department of Public Health (C-LL), Medical College, National Cheng-Kung University, Tainan; Department of Gynecology (PW); Department of Psychiatric (L-CH), Ditmanson Medical Foundation Chia-Yi Christian Hospital, Chia-Yi; Department of Pediatrics (SC-CC), School of Medicine, Taipei Medical University, Taipei; and Department of Emergency (F-SL), Ditmanson Medical Foundation Chia-Yi Christian Hospital, Chia-Yi, Taiwan.

## Abstract

The aim of the study was to investigate the epidemiology and risk factors of acute urinary retention (AUR) during pregnancy.

We included all cases of pregnancies with AUR reported in Taiwan's Longitudinal Health Insurance Database from January 1, 1998, to December 31, 2011. Cases of AUR onset 1 day before delivery were excluded. The Cochrane-Armitage trend test and logistic regression analysis were used to evaluate the age distribution and types of deliveries of pregnant women. Chi-square tests and Fisher's exact test were performed to examine the association among all covariates. The odds ratios (OR) and 95% confidence intervals (CI) were estimated.

We identified 308 cases of AUR in 65,490 pregnancies. The risk of AUR during pregnancy was 0.47%. The peak incidence occurred between the 9th and 16th gestational weeks. Patients who experienced preterm delivery exhibited the highest risk for AUR (2.18%). Those with post-term delivery had the second highest risk (0.46%), and patients with a normal delivery exhibited the lowest risk (0.33%). Compared with normal delivery, preterm delivery carried a higher risk of AUR (OR: 6.33, 95% CI: 4.94–8.11). The AUR risk was higher for patients with advanced maternal age (>35 years old) than it was for those in the younger group (< 20 years old) (OR: 2.62, 95% CI: 1.18–5.81). Within the normal delivery group, higher incidences of urogenital infection, gestational diabetes mellitus, previous abortion, abnormal pelvis, disproportion, and endometriosis were noted in women with AUR than in those without AUR (all *P* values <0.05).

Women with advanced maternal age and those who experienced preterm delivery had an increased risk for AUR. The peak incidence of AUR in normal pregnancies occurred between the 9th and 16th gestational weeks. Urogenital infection, gestational diabetes mellitus, previous abortion, abnormal pelvis, disproportion, and endometriosis were associated with AUR in women who underwent a normal delivery.

## INTRODUCTION

Acute urinary retention (AUR) is defined as a sudden and painful inability to voluntarily void urine.^1,2^ It is common in elderly men but unusual in women. Because pregnant women are relatively young, AUR during pregnancy is rare and typically goes unnoticed. However, AUR during pregnancy is not only an unpleasant experience but also a possible early sign of dire consequences, such as acute renal failure, spontaneous abortion, lifelong bladder dysfunction, and bladder rupture.^3,4^ For example, 2 women who presented with AUR in the second trimester were ultimately diagnosed with incarcerated uterus. One woman developed acute renal failure, and the other woman had bladder dysfunction after delivery.^3^ If AUR is well managed during its occurrence, subsequent complications may be avoided. Therefore, the early recognition and treatment of AUR is crucial to ensuring a normal pregnancy and avoiding potential complications.

The AUR during pregnancy occurs in all trimesters of gestation but is most often observed between the 10th and 16th gestational weeks. A retroverted uterus, uterine leiomyoma, an incarcerated fibroid uterus, ectopic pregnancy, lower genital infections, pelvic inflammatory disease, and lumbar disk diseases are all possible causes of this condition.^3–9^ Most of the reported AUR cases during pregnancy are self-limited and require either intermittent catheterization or short-term Foley catheter placement. However, some cases may require more aggressive interventions, such as manual reduction, amnioreduction, or surgical exploration.^3^

All previous studies of AUR during pregnancy have consisted of case reports or case series studies.^3–9^ To date, no population-based epidemiologic studies of AUR in pregnancy have been conducted. This study aimed to investigate the epidemiology and potential risk factors of AUR during pregnancy using a nationwide population-based database.

## PATIENTS AND METHODS

### Study Population and Data Source

The data were extracted from Taiwan's Longitudinal Health Insurance Database (LHID2000). The LHID2000 was established by the National Health Research Institute and includes original claims data of 1,000,000 patients who were randomly sampled from the National Health Insurance Research Database (NHIRD) in 2000. The representativeness of the LHID2000 has been validated by the National Health Research Institute. No significant gender or age distribution differences were observed between the LHID2000 and the NHIRD.

The LHID2000 provides information about both outpatient and inpatient visits. All the patient information was encrypted and anonymized. Using identification numbers, we could trace and analyze records from different clinical visits, hospital admissions, prescriptions, and medical procedures received, as well as demographic information (i.e., age, gender, and area of residence). Each record contains diagnostic fields coded according to the International Classification of Diseases, 9th Revision, Clinical Modification (ICD -9-CM). This study was approved by the Institutional Review Board (IRB) of Ditmanson Medical Foundation Chia-Yi Christian Hospital (IRB No: 104003). Due to the encryption of personal identification information, this study was exempt from full review by the IRB.

### Study Design and Inclusion Criteria

This study was designed as a retrospective, population-based, case cohort study to investigate cases of AUR during pregnancy between January 1, 1998, and December 31, 2011. All pregnancies with AUR, except abortions, were included. We excluded those pregnancies with AUR events that occurred 1 day before delivery to avoid the effects of anesthesia. We investigated the potential risk factors that contributed to AUR in women with a normal delivery, including urinary tract infection (UTI); inflammatory disease of the female pelvic organs, cervix, vagina, and vulva; and genital herpes. These factors, which all occurred within 1 week before the AUR onset, were recorded.

Moreover, we recorded the diabetes mellitus (DM), gestational DM and gyneco-obstetric histories of the patients, including information regarding leiomyoma, previous abortion, ectopic pregnancy, previous delivery, abnormal pelvis, multiple gestation, malposition/malpresentation, disproportion, hydra- or oligohydramnios, endometriosis, and dysmenorrhea.

### Definition

AUR was identified if a diagnostic code (ICD-9-CM code: 788.2) appeared with a charged fee for Foley catheterization during pregnancy. Only the first AUR event that occurred during the pregnancy period was included. Study subjects were excluded if they had a prior history of AUR diagnosis or Foley catheterization during the 3 months before the first AUR diagnosis during pregnancy.

The pregnancy period was determined according to the estimated number of gestational weeks and delivery date. The estimated number of gestational weeks was defined as follows: preterm delivery (ICD-9-CM code: 644.2), 30 weeks; normal delivery (ICD-9-CM code: 650), 39 weeks; post-term delivery (ICD-9-CM code: 645.1 and 645.2), 40 to 42 weeks.

### Statistical Analysis

The risk of AUR during pregnancy was estimated by dividing the number of pregnant women with AUR during pregnancy by the total number of deliveries. To examine the association of the risk of AUR during pregnancy with maternal age and the gestational period, logistic regression analysis was performed to estimate the crude odds ratio (OR) and 95% confidence intervals (CI). The Cochrane–Armitage method was used to test for trends. Chi-square tests and Fisher's exact test were performed to compare the frequency of potential risk factors between women with and without AUR during pregnancy. A *P* value of <0.05 was considered to be statistically significant. Data management and analyses were performed using the SAS/STAT^®^ software, version 9.3 for Windows (SAS Institute Inc., Cary, NC).

## RESULTS

### Association of AUR With Maternal Age and Delivery Pattern

A total of 308 cases of AUR were identified among 65,490 pregnancies during the study period. The age and delivery type distribution of these patients are listed in Table 1. The risk of AUR increased with maternal age. The risk of AUR was notably higher in pregnant women >35 years than in those <20 years old (OR: 2.62, 95% CI: 1.18–5.81).

**TABLE 1:**
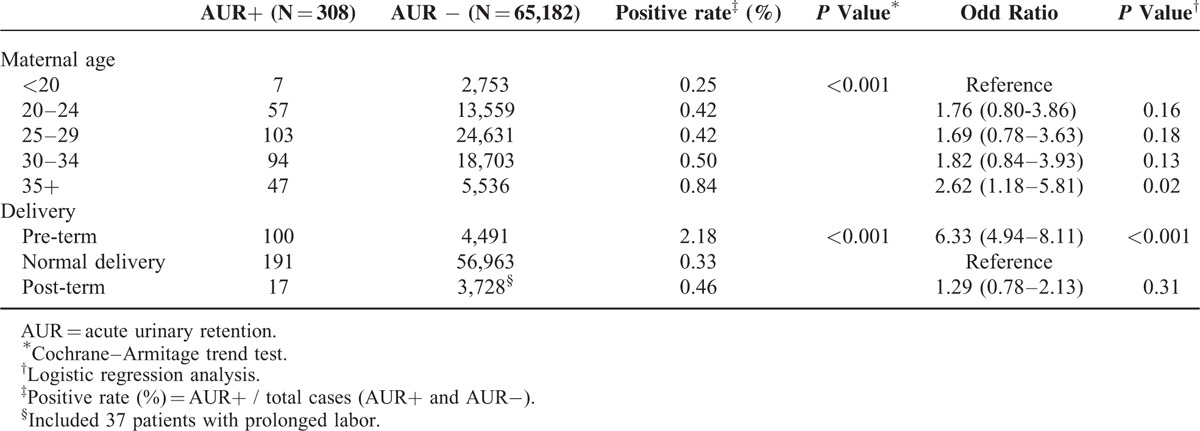
Age and Delivery Distribution in Pregnant Women With and Without AUR (n = 65,490)

As shown in Table 1, the risks of AUR were 2.18%, 0.33%, and 0.46% for women who underwent preterm delivery, normal delivery, and post-term delivery, respectively. Compared to normal delivery, preterm delivery was significantly associated with AUR, with an odds ratio (OR) of 6.33 (95% CI: 4.94–8.11). The OR of AUR was slightly higher for patients with post-term delivery than for those with normal delivery (OR 1.29, 95% CI: 0.78–2.13). Moreover, 1 female experienced AUR during both of her pregnancies. This information was not included in the tables.

### AUR Incidence According to Gestational Age

**FIGURE 1:**
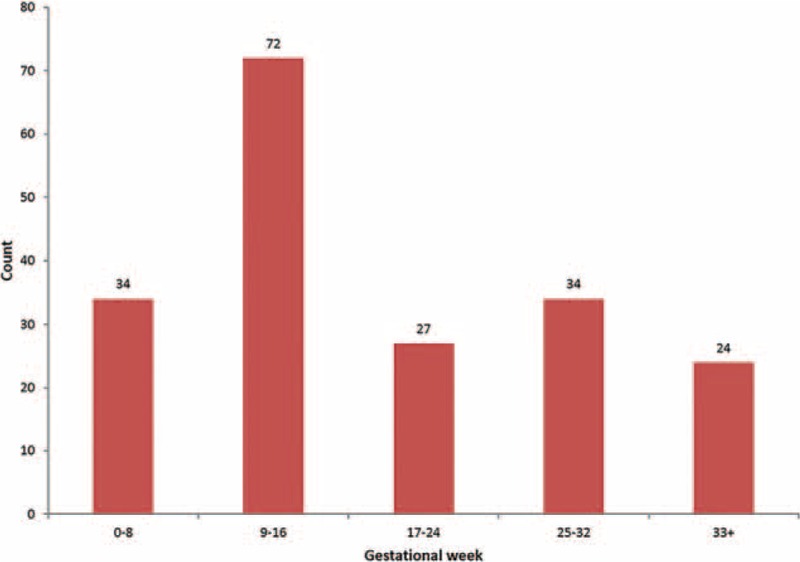
Frequency of the first AUR event in normal pregnancies by 8-week gestational intervals (n = 191). AUR = acute urinary retention.

We plotted the frequency of the first AUR event in women with normal pregnancies according to the number of gestational weeks, in 8-week intervals, in Figure 1. The number of initial AUR events during the 9th to 16th gestational weeks was at least 2-fold that observed during all other gestational periods.

### AUR-Associated Diseases

Table 2 shows the association of several related diseases with the risk of AUR during a normal pregnancy. As shown in Table 2, the incidences of gestational DM; UTI; inflammation of the pelvis, cervix, vagina, and vulva; genital herpes; previous abortion; abnormal pelvis; disproportion and endometriosis were significantly increased in the women with AUR compared with the women without AUR. In contrast, the women without AUR during pregnancy were more likely to have undergone previous deliveries (29.2% vs 17.2%).

**TABLE 2:**
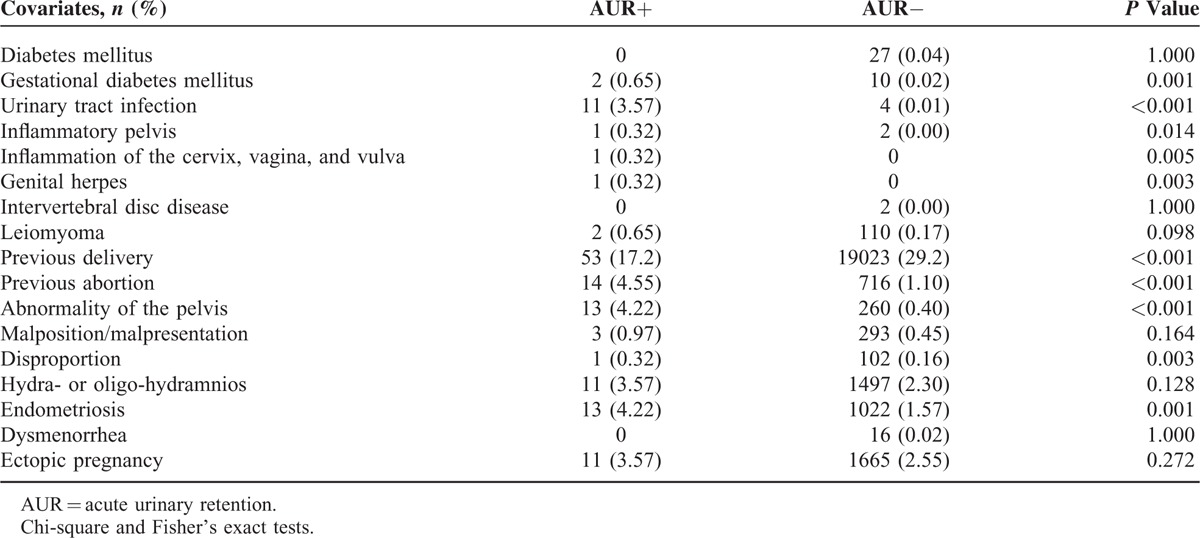
Covariate Comparisons Between the AUR and Non-AUR Groups in Women With a Normal Delivery

## DISCUSSION

This was a nationwide population-based epidemiological study. A total of 308 AUR cases were identified, corresponding to a risk of 0.47% among all pregnancies. Maternal age >35 years and preterm delivery were significantly associated with AUR. The most common period for the occurrence of AUR during normal pregnancies was from the 9th to 16th gestational weeks. In women with a normal delivery, urogenital infection, previous abortion, and an abnormal pelvis were risk factors associated with AUR.

Van der Linden *et al* reported an incidence of AUR in women of 0.07 per 1000 women per year.^10^ The causes of AUR in women can be broadly categorized as infective, pharmacological, neurological, anatomical, myopathic, and functional.^11^ Kavia et al investigated 247 women (mean age 35 years) with complete or partial urinary retention. They found that the most common cause of urinary retention in these young women was a primary disorder of sphincter relaxation (Flower's syndrome).^12^ However, detailed bladder assessments of pregnant women with AUR are rarely conducted due to ethical and medical objections. Moreover, the results of urodynamic studies during pregnancy are often contradictory and have not been clinically helpful.^13^

The most studied components of AUR during pregnancy are anatomical factors. Extrinsic bladder neck or urethral compression due to an impacted and enlarging uterus has been postulated as the pathogenic mechanism of AUR during pregnancy.^3,4,6–8,14^ Weekes *et al* reported that ∼11% of pregnancies involved a retroverted uterus, and 1.4% of these cases resulted in AUR.^14^ In a study by Yang *et al*, sonographic evidence of bladder neck compression by a displaced cervix was found in 5 pregnant women with AUR and a retroverted gravid uterus.^7^ The retroverted gravid uteruses usually rotated spontaneously to an upward position before the 14th gestational week. As the compression was relieved, micturition returned to a normal state.^3^ This finding may explain why some AUR cases during pregnancy are temporal and self-limited. One case-series study from Pakistan investigated 15 pregnant women with AUR and revealed that 66.6% of AUR cases occurred during the 1st trimester, whereas 33.3% occurred in the 2nd and 3rd trimesters.^8^ The timing of the peak incidence of AUR in our study was consistent with that of previous studies.^3–8^

However, a retroverted uterus could not fully explain AUR in pregnancy. A retroverted uterus is present in only 11.2% to 15% of pregnancies.^3,7,14^ Factors such as pelvic adhesions, congenital pelvic abnormality, posterior uterine wall leiomyoma, and endometriosis might prevent the uterus from entering the abdominal cavity.^3,4,6^ In our study, previous abortion, pelvic abnormality, disproportion, and endometriosis were associated with AUR in women with a normal delivery. Although the number of included cases in our study was small, the statistical associations were significant. Our results implicated adhesion and structural factors in the development of AUR.

Interestingly, we identified more women with a previous delivery history in the non-AUR group compared with the AUR group (Table 2). This finding suggested a protective effect of a previous delivery against AUR, which was not reported in previous studies.^3–8^ Some studies have compared lower urinary tract symptoms (LUTS) between primiparous and multiparous women.^15–20^ All these studies have indicated that parity is a significant risk factor for stress urinary incontinence. Stress urinary incontinence involves uncontrollable urine leakage, which is very dissimilar to urinary retention. We reasoned that the gravid mechanical distension effect on the pelvic area, abdominal cavity, and urethra might decrease the risk of AUR.

LUTS is a typical finding in the elderly, in both males and females, due to the physiological changes associated with ageing. In a study by Madersbacher et al, urodynamic changes with aging were observed in women. A significant decrease in the maximal flow rate (*Q*_max_), voided volume, and bladder capacity but increased post-voiding residual urine were noted.^21^ The prevalence rate of LUTS increases with gestational age.^17,18,20^ In our study, we also showed that advanced maternal age (> 35 years old) was a significant risk factor for AUR.

Comorbidities and acute illness may adversely affect urinary tract function. However, pregnant women are relatively young and typically have fewer comorbidities. In our study, 27 pregnant women had DM. According to the literature, the incidence of type 1 DM has been reported to be low in Taiwan, ∼5 per 100,000,^22^ and the most recent prevalence of DM in 20 to 39 y/o females was 0.36 to 0.52% in Taiwan.^23^ In addition, this study investigated the history of diabetes before the AUR event. More than half of the AUR events occurred before the 24th gestational week, which is earlier than the routine test for gestational DM conducted between the 24 to 28th gestational weeks. These reasons may explain the relatively small number of DM cases in this study.

Nevertheless, we found that UTIs, inflammation of the pelvis and genital organs, and genital herpes were associated with AUR in women with a normal delivery. These were all acute illnesses that could possibly cause AUR. Infection factors cannot be neglected when considering the etiology of AUR during pregnancy. One study conducted in Pakistan analyzed 15 women with AUR during pregnancy and demonstrated that 4 (26.7%) patients had a lower genital tract infection.^8^ However, the infection rate in this study was extremely low. One potential reason is that in our society, pregnant women are often reluctant to take medicines because of the fear of fetal toxicity. They typically avoid visiting physicians unless the illness is clearly serious. In addition, we only recruited women with inflammatory diseases diagnosed within 1 week before AUR onset. Therefore, an underestimation of UTIs and other inflammatory diseases was possible. Moreover, women with preterm delivery had more gynecological or fetal illnesses than women with a normal pregnancy; in our study, the risk for AUR was significantly higher in these women. Further studies focusing on preterm delivery are necessary to elucidate the association between AUR and preterm delivery.

Some limitations were encountered in this study. First, pregnant women delivering between the 22nd and 37th weeks were classified as having a preterm delivery; those delivering between the 37th and 40th weeks were classified as having a normal delivery, and those delivering between the 40th and 42nd weeks were classified as having a post-term delivery. The exact gestation period of each pregnant woman was not available from the NHIRD. However, by reviewing the maternal delivery data from our own hospital, we determined that the median gestational week definition used in the present study was appropriate. Therefore, the period definition bias was reduced. Second, due to the limited number of cases of preterm, post-term, and prolonged term deliveries, we could only analyze covariates in women with a normal delivery. Further large-scale studies may be necessary to explore these associations in other types of deliveries. Third, we did not include a medicinal effect on AUR because the pregnant women were relative young and reluctant to take medicine for the fear of drug effects on the fetus. Thus, potential medicinal effect on AUR was ignored in this study.

## CONCLUSIONS

AUR during pregnancy is an uncommon and multifactorial disease. The risk of AUR during pregnancy is 0.47%. Women with advanced maternal age (> 35 years-old) and those who experience preterm delivery have an increased risk for AUR. The peak incidence of AUR onset in normal pregnancies occurred between the 9th and 16th gestational weeks. To minimize AUR-related complications and identify high-risk pregnancies, the early diagnosis, appropriate treatment, careful evaluation, and close follow-up of AUR during pregnancy are necessary.
